# Imagined Self-Motion Differs from Perceived Self-Motion: Evidence from a Novel Continuous Pointing Method

**DOI:** 10.1371/journal.pone.0007793

**Published:** 2009-11-11

**Authors:** Jennifer L. Campos, Joshua H. Siegle, Betty J. Mohler, Heinrich H. Bülthoff, Jack M. Loomis

**Affiliations:** 1 Max Planck Institute for Biological Cybernetics, Department of Human Perception, Cognition and Action, Tübingen, Germany; 2 Department of Brain and Cognitive Engineering, Korea University, Seoul, Korea; 3 Department of Psychology, University of California Santa Barbara, Santa Barbara, California, United States of America; University of Regensburg, Germany

## Abstract

**Background:**

The extent to which actual movements and imagined movements maintain a shared internal representation has been a matter of much scientific debate. Of the studies examining such questions, few have directly compared actual full-body movements to imagined movements through space. Here we used a novel continuous pointing method to a) provide a more detailed characterization of self-motion perception during actual walking and b) compare the pattern of responding during actual walking to that which occurs during imagined walking.

**Methodology/Principal Findings:**

This continuous pointing method requires participants to view a target and continuously point towards it as they walk, or imagine walking past it along a straight, forward trajectory. By measuring changes in the pointing direction of the arm, we were able to determine participants' perceived/imagined location at each moment during the trajectory and, hence, perceived/imagined self-velocity during the entire movement. The specific pattern of pointing behaviour that was revealed during sighted walking was also observed during blind walking. Specifically, a peak in arm azimuth velocity was observed upon target passage and a strong correlation was observed between arm azimuth velocity and pointing elevation. Importantly, this characteristic pattern of pointing was not consistently observed during imagined self-motion.

**Conclusions/Significance:**

Overall, the spatial updating processes that occur during actual self-motion were not evidenced during imagined movement. Because of the rich description of self-motion perception afforded by continuous pointing, this method is expected to have significant implications for several research areas, including those related to motor imagery and spatial cognition and to applied fields for which mental practice techniques are common (e.g. rehabilitation and athletics).

## Introduction

Self-motion perception refers to the subjective experience of moving though space, which, under most natural conditions, occurs when a person is actually moving. Typically, the availability of several sources of information allows self-motion to be perceived in an effortless and obligatory manner. These sources include visual, proprioceptive, inertial, and cognitive inputs. Evaluating and precisely characterizing self-motion perception under different sensory conditions has been important for a wide variety of research questions. In particular, attempts to define and quantify self-motion perception have been prominent when investigating humans' abilities to use particular sensory cues in isolation. This has included passive transport experienced via inertial cues alone, dynamic visual information alone, and walking in the absence of vision. In a separate research area, investigators have been interested in self-motion perception during purely imagined movements. The question of whether the processes involved in imagery share a common underlying mechanism with direct sensory perception has been debated for several decades [1]–[3]. However, this has not been an issue that has received as much attention in research areas investigating full-body motion through space [4]–[10].

In this study, a novel continuous pointing method developed by our group was used to measure and precisely characterize aspects of self-motion perception during actual movement and imagined movement through space. This method simply requires participants to view a target and point continuously towards it as they move past it, or imagine moving past it, along a straight, forward trajectory. As will be described in detail below, by measuring changes in the pointing direction of the arm, this method is able to infer instantaneous changes in perceived/imagined location, velocity, and acceleration. The detailed characterization of self-motion perception during actual movement is then compared to the pattern of pointing behaviour observed during imagined self-motion. Continuous pointing is ideally suited for uncovering the covert, online processes that occur during imagined movements.

### Actual Self-Motion Perception under Reduced-Cue Conditions

Much work has been conducted to understand what sensory information is necessary and/or sufficient for navigation and spatial updating. For instance, there has been an extensive body of research investigating how the inertial information transduced by the vestibular organs (the otoliths and the semicircular canals) can be used to update one's position in space when moving along simple, linear and rotational trajectories [11]–[19], and when travelling along more complex paths [20]–[22]. The capacity of using optic flow alone to accurately estimate different aspects of self-motion (e.g. distance, speed, heading) has also been investigated using similar tasks [23]–[29]. Characteristics of visually-induced perceived self-motion, referred to as “vection”, have also been explored [30]–[32].

Important to the current experiment are studies that have examined conditions in which only idiothetic cues (e.g., inertial and proprioceptive cues) remain available during walking when vision is absent (i.e. during blindfolded walking). A widely-used method to investigate spatial updating has been the “blind-walking task” [19], [33]–[40]. In this task, observers view a target positioned at some distance, close their eyes and walk to the location of this target in the absence of vision. A variant of this task is triangulation by walking or pointing, which involves viewing a target, walking on an indirect path and, when prompted, walking or pointing toward the updated target [35], [41]. Still another variant is the blind walking/gesturing task [42], which involves walking without vision to the updated target and then gesturing with the hand to indicate its location in space, including its height.

Loomis and colleagues have proposed that when visual feedback is removed after an initial exposure to a visual target, a “spatial image” of the perceived target location is maintained [43]. Consequently, when moving in darkness, target-relative egocentric positions can be tracked using the updated position of the observer relative to the spatial image. During spatial updating tasks, performance errors can be attributed to errors in initially perceiving the location of the target, errors in perceiving self-motion, or errors in mentally updating the spatial image during responding [43]. It is therefore important to differentiate the various sources of error and, if possible, to quantify them.

Overall, it is clear that there have been great strides in understanding many different aspects of self-motion perception under a wide range of sensory/motor conditions. That said, there are several limitations associated with the types of measurements that have traditionally been used and much that remains to be explored (see also [17]). The continuous pointing method used here provides a more detailed description of perceived self-motion when walking in the absence of vision. In addition, the pattern of responding that is observed under blindfolded walking conditions serves as a useful comparison by which to assess the internally-based self-motion representations that occur during other reduced cue conditions, specifically imagined self-motion.

### Imagined Self-Motion Perception

Imagined movement, also described as motor imagery, refers to the mental simulation of an actual, physical movement. The imagined viewpoint is that associated with the observer's subjective experience from a first-person perspective rather than observing their own movements from an external perspective, or observing someone else's movements. Investigations evaluating the similarity of the mechanisms underlying imagined movements and actual movements have been conducted using a variety of different paradigms.

Evidence supporting the idea that similar mechanisms sub-serve actual and imagined movements has been provided by neuroimaging studies [1], [44], [45], autonomic response measurements [6], [44], patient populations [46], motor imagery questionnaires [47], [48] and chronometric or temporally-based comparison tasks [4], [7], [8], [10], [49]. For instance, using fMRI, similar patterns of activation have been found when an observer is both imagining performing and physically performing a particular movement of the extremities [44]. Considering that it is difficult to acquire external feedback from internal processes such as those involved in motor imagery, imaging studies are often thought to provide unique access to covertly simulated actions (although this approach has also been criticized [50]). Others have looked at objective autonomic response measures, finding that heart rate and respiration rates increase proportionally in response to imagined movements of different intensities [5], [44].

Perhaps the most popular methods of assessing the shared characteristics of imagined and actual movement have been chronometric or time-based tasks. These tasks typically compare the time that it takes to imagine performing an action to the time that it takes to actually perform that same action [8], [51], [52]. For instance, it has been shown that it takes the same amount of time for an observer to imagine writing a phrase than it does to actually write the same phrase [51]. Looking at similar chronometric tasks in patients with unilateral brain lesions has also demonstrated that the patient's physical limitations are reflected in their imagined movement performance [46]. For instance, after damage to the left hemisphere, patients take longer to both actually write and to imagine writing with their right hand. Finally, in order to evaluate an observer's imagery ability, several questionnaires have been used including, the Movement Imagery Questionnaire [47] and the Vividness Movement Imagery Questionnaire [48]. While each of these approaches provides important insights, it is not uncommon for almost identical approaches to result in inconsistent findings. Further, the similarity between motor imagery and actual movement can depend on the population being studied (e.g. athletes versus non-athletes) and the nature of the task [2], [52].

Of all the motor imagery tasks that have been studied, only a small subset has included conditions involving full body self-motion through space. Of those, almost all of them have used chronometric tasks. For instance, several investigators have demonstrated that, when asked to imagine walking to a previously viewed visual target, the duration of the imagined response is almost identical to the duration of the actual walked response [8], [49]. Others have recently reported that imagined walking times are in fact faster than the time it takes to physically walk blindfolded to a previously viewed target [4], [7], [9]. Plumert et al. (2004) reported faster imagined walking times compared to actual sighted walking, but almost identical imagined walking times and blind-walking times. In almost all cases, however, the variability of responding is often higher for imagined response times compared to actual walking times. Kunz et al. (in press) also report that the differences between actual and imagined time-to-walk are reduced if a behaviourally relevant action is being performed during imagined walking (i.e. walking in place), but not when an inconsistent action is being performed (i.e. hand waving). Further, it has been shown that during conditions under which environmental constraints affect actual walking speed (i.e. walking along a narrow vs. a wide path), imagined walking times are affected in the same way as actual movements [10]. However, when imagining an object moving along the same paths (rather than imagining self-motion), physical path characteristics do not affect timing estimates [10].

Perhaps one of the reasons that studies on imagined self-motion have been restricted to chronometric tasks is the difficulty or even the impossibility of using alternative methods. For instance, current neuroimaging techniques cannot be used during full-body self-motion. While some have looked at patterns of neural responses for different types of imagined locomotor behaviours like swimming [53], walking, and running [54], there is no available comparison for patterns of neural responding during the actual behaviour.

There are also several limitations to what can be directly inferred from the results of chronometric tasks alone. Specifically, certain strategies could allow the participant to accurately estimate the time that it would take to perform an action without using the same underlying mechanism that is used during the actual movement. Many investigators have referred to the fact that tacit knowledge can be used to effectively perform these types of tasks. Therefore, it is difficult to differentiate between a vivid experience of self-motion during imagined movements and simply using strategies based on knowledge that has been obtained through one's interactions with the world (e.g., knowing how long each step takes and how far it moves one forward). While certain manipulations may make tacit knowledge more difficult to use and a higher reliance on more implicit motor processing more likely [4], [7], [10], the precision of what can be revealed about the characteristics of imagined movements remains limited.

In general, limitations of imagined self-motion methods include difficulties in dissociating the effects of top-down strategies from automatic obligatory motor processing, a reliance on subjective reporting, higher susceptibility to effects of experimenter instructions (e.g. prescribed walking speed), and the significant information loss that comes from relying on discrete post-hoc response measures. Overall, it currently remains unknown whether the mechanisms underlying spatial updating during actual self-motion and imagined self-motion overlap, or whether the processes can be completely dissociated.

### Continuous Pointing as a Method of Measuring Self-Motion Perception

Continuous pointing overcomes several of the challenges faced by past measures of self-motion perception and motor imagery by allowing for the identification of perceived/imagined location and hence perceived/imagined self-velocity instantaneously in real-world units. It is also a highly intuitive task that does not require participants to explicitly describe properties of their perceived/imagined self-motion. In fact, observers are not even asked to attend to any particular feature of their movement, such as speed or distance travelled. This means that the derived estimates of speed and distance are less likely to be affected by the participant's expectations or biases and are more likely to reflect natural, intuitive movement-related responses. Because the data are collected online as the pointing movement is being performed, this also reduces the potential for memory-related artefacts that can arise from post-hoc judgments or two-interval forced choice tasks. Most importantly, the arm movements observed during natural, sighted walking have a distinct pattern that can be compared to those during self-motion under other reduced cue conditions. First, a peak in arm azimuth velocity occurs at the point at which the observer passes the target. Second, for targets on the floor, arm azimuth velocity is highly correlated with pointing elevation. Specifically, peak arm azimuth velocity (i.e. upon target passage) corresponds closely with the point of lowest arm elevation. The key comparison will be to evaluate whether the highly coordinated spatial and temporal structure of arm movements observed during sighted walking will also be observed during blind-walking and, most interestingly, during imagined walking. This is the first time that this novel method has been used to directly answer these questions. Based on past studies, it is clear that some overlap exists between the processes involved in imagined and actual movements. However, few would argue that the processes underlying each are identical. Therefore, rather than describing the broad similarities that exist between the two, it is now important to develop sensitive measures that will allow us to more precisely define the ways in which they differ.

This experiment took place in a large, well-lit, fully tracked, free-walking space, 12 m×15 m in size. Participants' head positions and the position of a handheld-pointer were tracked using an optical tracking system (16 Vicon MX13 cameras). The visual target consisted of a solid white Styrofoam ball, 16 cm in diameter. The target was either positioned on the ground or elevated at each individual participant's shoulder height and was always located in the centre of the room.

**Figure 1:**
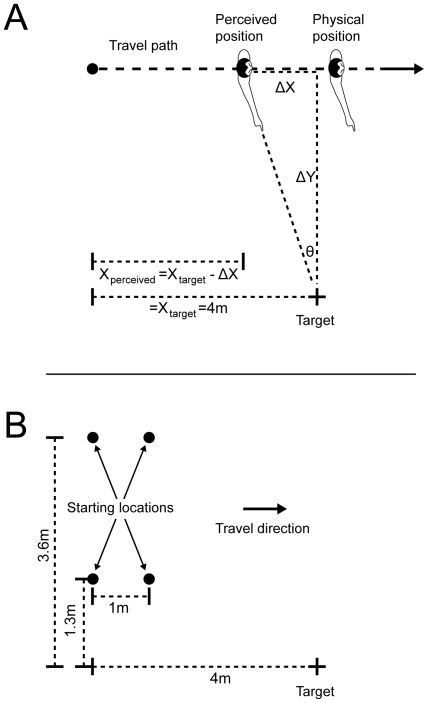
General procedure. A) Participants began each trial at one of several target-relative starting locations. After viewing the target, they moved or imagined moving past it along a straight travel path. While they moved, they pointed continuously to the spatial image of the target (or to the actual target during sighted walking). For the actual self-motion trials (SW and BW), based on arm angle and the known value of *y*, we computed *x*, or perceived distance from the target, throughout each movement trial. As shown here, there may be a discrepancy between a participant's perceived and actual location which would indicate a misperception of self-motion. The extent of arm movements used for analyses was within a comfortable and unconstrained motion range. B) Four different target-relative starting locations were used for this task.

During actual walking conditions, the general task required participants to view the target and then point continuously towards the remembered location of the target (spatial image) as they moved past it along a straight, forward trajectory (Figure 1a). Once they had walked at least two meters past the target, participants received a verbal cue from the experimenter to stop moving and pointing. To ensure that no feedback about performance was possible, participants were directed to immediately lower their arm and keep their eyes closed until they were brought to the next starting position via an indirect route. The starting positions relative to the target were varied on each trial and were tested from different areas of the room (Figure 1b). Participants were initially positioned 3 m or 4 m from the point of nearest target approach along the line of travel (resulting in a travelled distance of 5 m or 6 m) and the line of travel was either displaced 1.3 m or 3.6 m to the left of the target. During the imagined walking condition, the task was identical, except that participants first viewed the target, closed their eyes, and continuously pointed at the remembered position of the target as they imagined walking past it on a straight, forward trajectory.

By assuming that the participant's arm pointed toward the spatial image from the perceived or imagined self-position as they intended, the internally-represented self-position of the participant at each point in time could be obtained. For each trial during the actual self-motion conditions, each sampled pointing angle was converted to an estimate of perceived location. As shown in Figure 1a, the perceived X coordinate is given by the equation X*_perceived_*  =  X*_target_* − ΔX. Here ΔX  =  ΔY tan *θ*, where *θ* equals the recorded azimuth of the arm (i.e., rotation around a vertical axis) and ΔY is the distance between the target and the travel path. Thus, by tracking the pointing device, a continuous sampling of estimated perceived locations along each trajectory was obtained. Differentiating perceived location with respect to time yielded a measure of continuous perceived velocity. Velocity data were low-pass filtered to reduce noise (first-order Butterworth filter, cutoff frequency of 1 Hz).

Considering that we were unable to determine the actual distance travelled during imagined self-motion, a different analysis was used. To compare the internally-represented self-motion trajectory in the imagined condition to perceived self-motion in the actual movement conditions, we assessed how instantaneous arm velocity varied systematically with arm azimuth. Arm velocity was normalized by dividing each trial by the peak velocity across the entire trial. Considering that peak velocity should be observed upon target passage when arm azimuth is zero, the extent to which it deviates from this provides us with insight into the characteristics of perceived/imagined self-motion.

A conversion of the pointing data into the perceived or imagined location is based on three supported assumptions [17]: (1) visual perception of the initial target location is accurate, as would be expected given the full-cue viewing conditions of the current experiments [43], (2) the direction vector of perceived/imagined self-motion is initially aligned with the straight, forward trajectory and exhibits little or no veer [55], and (3) the act of pointing with the arm does not introduce any additional systematic error beyond that associated with perceived or imagined self-motion.

Each participant performed the continuous pointing task for a series of conditions, three of which are reported here. First, in order to establish baseline pointing behaviour under full-cue conditions, participants viewed the target from the starting position and then continuously pointed to it as they walked past it with their eyes open (Sighted Walking: SW). This condition was not intended to measure perceived self-motion per se, but rather to account for errors in the physical tracking measurement procedure and calculations. When the participant's eyes are open, accurate pointing toward a target is possible whether or not the participant experiences self-motion. Thus, pointing with the eyes open cannot be used to compute the participant's perceived position or perceived self-motion. Nevertheless, in order to simplify the exposition that follows, we use the same terminology of “perceived position” and “perceived self-motion” for the sighted walking condition as we use for the blind walking condition.

Second, in the Blind Walking condition (BW) participants first viewed the target and pointed to it from the starting position under full-cue conditions. When prompted by the experimenter, they closed their eyes and continuously pointed to the target as they walked past it. Third, in the Imagined Walking condition (IM), participants viewed and pointed to the target from the starting position under full cue conditions and subsequently closed their eyes and imagined walking forward on a straight path past the target. They continuously pointed at the spatial image of the target throughout the trial. Prior to the IM trials, participants were asked to walk around the target in a square formation without pointing in order to establish a natural walking speed that they could then mentally refer to during imagined walking. A subset of the participants completed the imagined walking first, while the remainder completed the imagined condition last. This was done to evaluate the effects of having experienced pointing during several actual self-motion conditions prior to the imagined self-motion trials.

## Results

### Perceived Self-Position and Self-Velocity during Actual Self-Motion

**Figure 2:**
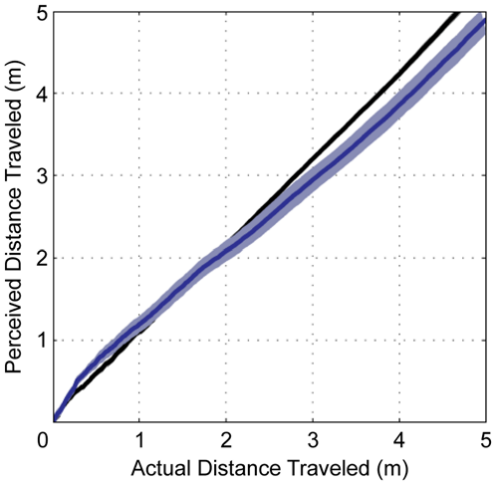
Perceived versus actual self-position. Average perceived self-position relative to actual position during SW (black line) and BW (blue line). The shaded areas represent plus and minus one standard error of the mean.

When comparing the patterns of arm azimuth across target height and target displacement, there were no differences between perceived self-position and self-velocity, therefore the data were collapsed for subsequent analyses of the actual self-motion conditions unless otherwise specified. First, participants' perceived position (calculated via arm azimuth) was compared to their actual position in space (calculated via the head tracking data) for the SW and BW conditions (Figure 2). In both conditions, average signed error scores were low (Mean across the two path lengths were: SW = 11.7 cm and BW = 17.0 cm) and did not differ significantly from each other (*t*(11) = −0.54, *p* = 0.60; paired samples t-test). This is particularly accurate considering that that the target itself was only 16 cm in diameter. Paired sample t-tests were also conducted to compare the average perceived location of participants upon target passage for the SW and BW conditions and demonstrate that there was no significant difference for either the 5 m path (*t*(11) = 0.30, *p* = 0.71) or the 6 m path (*t*(11) = 0.90, *p* = 0.39).

**Figure 3:**
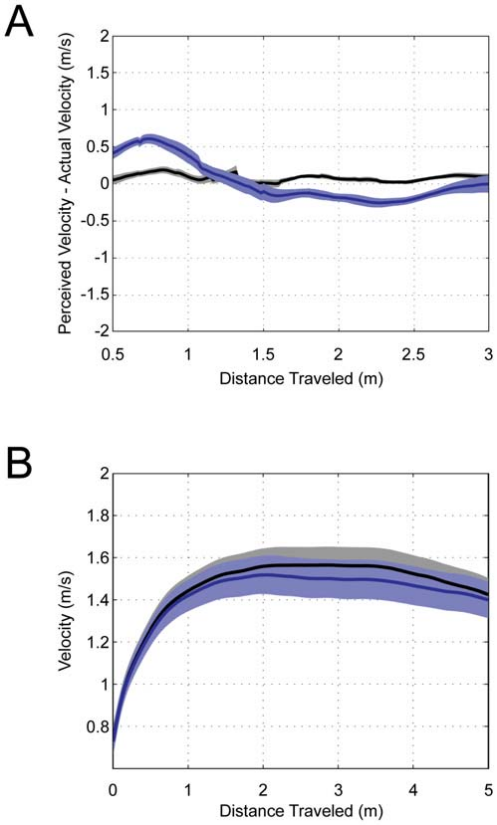
Perceived versus actual self-velocity. A) Signed velocity error values (perceived minus actual self-velocity) during SW (black line) and BW (blue line). B) Absolute head velocities for the SW (black line) and BW (blue line) conditions. The shaded areas represent plus and minus one standard error of the mean.

Differentiating perceived location with respect to time yields perceived velocity for each of the actual self-motion conditions. The average perceived velocities were 1.56 m/s (±0.29) for the SW condition and 1.52 m/s (±0.31) for the BW condition. There was no significant difference between average perceived self-velocity and actual self-velocity for SW or for BW (collapsed across the two path lengths; Figure 3a). There was also no significant difference between actual walking velocity for the SW and BW conditions as evidenced through the head-tracking data (Figure 3b).

### Actual Self-Motion versus Imagined Self-Motion

All of the following analyses on arm azimuth data were conducted using data from the nearest target distance trials (1.3 m displacement) due to the fact that arm azimuth measures are more sensitive for targets within closer proximity. Also, there were no differences between the 5 m and 6 m distance trials and therefore the data were collapsed.

**Figure 4:**
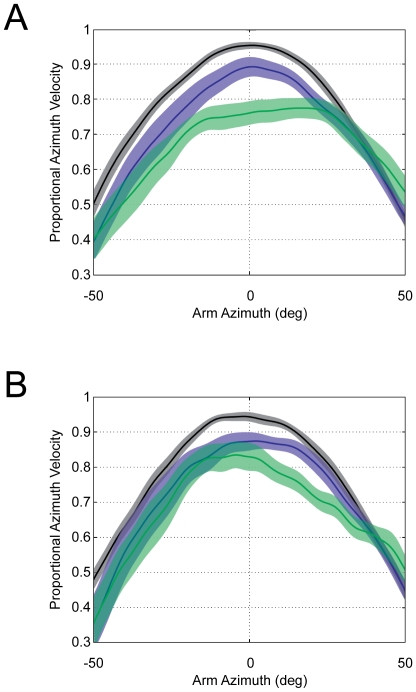
Imagined versus actual self-motion: comparison of peak arm azimuth velocity. Percentage of maximum angular velocity at each degree of arm azimuth. For ideal pointing behaviour, peak arm velocity will occur at zero degrees of arm azimuth. This was true during both of the actual self-motion conditions (SW indicated in black and BW indicated in blue), but not during imagined self-motion (green line). The shaded areas represent plus and minus one standard error of the mean. A) The pattern of responding for the elevated target trials. B) The pattern of responding for floor level target trials.

In order to compare the imagined self-motion condition to the actual self-motion conditions, we evaluated the manner in which instantaneous arm velocity varied systematically with arm azimuth. Considering that the peak arm azimuth velocity should be observed upon target passage when arm azimuth is zero, the extent to which it deviates from this prediction provides us with insight into the characteristics of perceived/imagined self-motion. In this analysis we compared the percentage of the maximum arm azimuth velocity at zero degrees for each condition and each distance for both elevated targets (M: SW = 95%; BW = 89%; IM = 76%; Figure 4a) and floor-level targets (M: SW = 95%; BW = 87%; IM = 83%; Figure 4b). Paired sample t-tests indicate that for the elevated targets there was a significant difference between SW and BW (*t*(11) = 2.52, *p*<0.05) and between BW and IM (*t*(15) = −3.94, *p*<0.001). The same analyses on floor-level targets indicate a significant difference between SW and BW (*t*(11) = −3.17, *p*<0.05) and between SW and IM (*t*(10) = 2.91, *p*<0.05), but no significant difference between BW and IM *(t*(10) = −1.08 *p* = 0.3).

When looking at Figures 4a and 4b it becomes apparent that during the SW and BW conditions, which both involved actual self-motion, a characteristic pointing movement profile was observed. Specifically, arm azimuth velocity increased systematically upon target approach and reached a peak velocity close to arm azimuth values near 0°. For elevated targets the mean peak velocity for SW occurred at 2.5° and for BW at 1.8°. For floor-level targets the mean peak velocity for SW occurred at −1.15° and for BW at −2.52°. This same pattern was not observed during imagined self-motion, for which the peak velocity was reached either much later (IM elevated target = 15.2°) or earlier (IM floor target = −8.07°).

Further, whereas the average angular arm velocity changed systematically between −20° and +20° arm azimuth (i.e. just before and just after target passage) for both SW and BW conditions, it did not change as systematically in the IM condition. Instead, it either reached a plateau too early and remained there until deceleration (elevated targets, Figure 4a) or decelerated too rapidly after target passage (floor target, Figure 4b). Specifically, for both target heights in the IM condition, the maximum angular velocity at an arm angle of −20 degrees did not differ significantly from that at zero degrees (elevated target: *t*(15) = −1.97, *p*>0.05; floor target: *t*(10) = 1.37, *p*>0.05). This was not consistent with the actual self-motion conditions, in which case the velocities between −20 degrees and 0 degrees did, in fact, differ significantly; (SW, elevated target *t*(11) = −8.27, *p*<0.001; SW, floor target *t*(11) = 4.10, *p*<0.01) and (BW, elevated target *t*(17) = −4.11, *p*<0.001; BW, floor target *t*(11) = 2.39, *p*<0.05). The same pattern of results was also observed when comparing the maximum angular velocity at an arm angle of +20 degrees for elevated targets (IM:(*t*(15) = 0.38, *p* = 0.71); SW: (*t*(11) = −5.91, *p*<0.001); BW: (*t*(17) = −3.93, *p*<0.001), and for floor targets there was a significant difference in all three conditions (IM:(*t*(10) = 3.43, *p*<0.01.); SW: (*t*(11) = 5.21, *p*<0.001); BW: (*t*(11) = 2.47, *p*<0.05).

Finally, root mean square (RMS) values were used to calculate the extent to which the observed curves in each condition (SW, BW and IM) deviated from the ideal pointing curve. These were calculated between ±20° arm azimuth and were collapsed across the two distances. These error values indicated that the lowest RMS values were observed for SW responses (i.e. a closest fit to ideal pointing); floor = 0.08 (±0.03), elevated = 0.07 (±0.02). BW exhibited the second lowest RMS values; floor = 0.17 (±0.09), elevated = 0.18 (±0.13), and the highest RMS values were observed for the IM condition (i.e. the largest deviation from ideal pointing); floor = 0.24 (±0.10), elevated = 0.25 (±0.10).

### Arm Azimuth Velocity versus Arm Elevation

**Figure 5:**
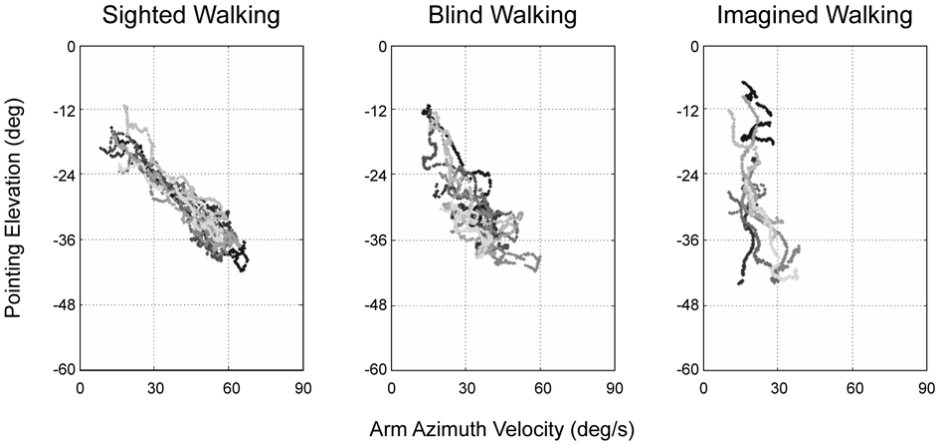
Correlation between pointing elevation and arm azimuth velocity. When pointing to the floor targets, the lowest arm elevation should be highly correlated with the fastest arm azimuth velocity (lowest elevation  =  fastest arm velocity). During both SW and BW, a high correlation was observed, whereas a much lower correlation was observed for IM. This figure includes the data for one representative participant. In the overall analysis, correlations were performed for each participant's data and averaged across all participants. The data indicated by the different tones of grey represent individual trials for one participant.

For the conditions in which participants pointed to the target on the ground, we were able to evaluate the extent to which changes in pointing elevation correlated with changes in arm azimuth velocity. Specifically, correct pointing would dictate that the lowest pointing elevation should co-occur with the highest arm velocity at zero degrees arm azimuth. Correlations were calculated for each trial and averaged across participants (See Figure 5 for scatter plots of a representative participant). The results indicate a negative correlation between arm elevation and arm azimuth velocity for all of the actual self-motion conditions, and a lower negative correlation for the imagined self-motion condition (Mean across path lengths r: SW = −0.91; BW = −0.60; IM = −0.48). The percentage of trials in which the correlations were significant was 100% for SW, 89% for BW, and 63% for IM.

### Effect of Performing Imagined Pointing before and after Actual Self-Motion

Six of the participants who completed all three conditions (SW, BW and IM) completed the imagined condition first (before any of the actual self-motion conditions), while another comparable six participants completed the imagined condition last (after having completed all other actual self-motion conditions). In order to evaluate whether the experience of pointing during self-motion immediately prior to the imagined pointing trials had any effect on imagined pointing performance, the patterns of responding in the two groups were compared.

**Figure 6:**
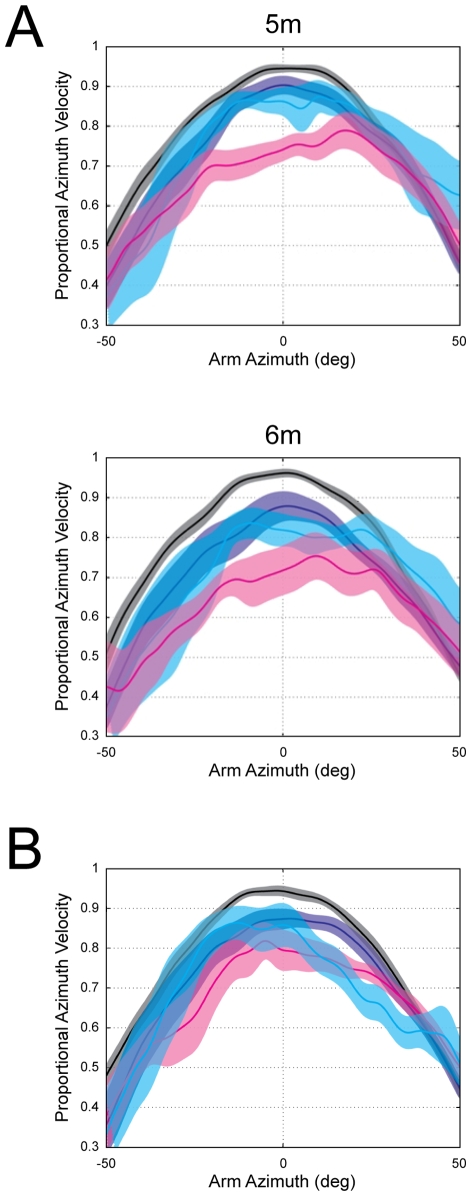
Difference between completing the imagined condition first versus last. Six of the participants completed the IM condition first, while another six completed it last. Here, the percentage of maximum angular velocity at each degree of arm azimuth is compared for participants who completed the IM condition first (magenta line) to the participants who completed it last (cyan line). Black lines represent SW and blue lines represent BW. A) For the elevated target trials, a significant difference was observed when comparing the IM first versus IM last trials for the 5 m path length but not for the 6 m path length. B) For the floor level targets there was no difference between IM first and IM last for either distance. The shaded areas represent plus and minus one standard error of the mean.

The difference between the percentage of maximum azimuth velocity at zero degrees of arm azimuth for the BW and IM conditions were compared for participants who completed the IM condition first versus last. Pointing responses for the elevated target trials were not significantly different when comparing IM first versus IM last conditions when collapsed across distances. However, when considering the 5 m and 6 m path lengths separately, different results were observed (Figure 6a). Specifically, a significant difference between IM first versus IM last was observed for the 5 m path length trials (*t*(9) = 2.74, *p*<0.05), but not for the 6 m path length trials (*t*(9) = 0.53, *p* = 0.61). This is likely explained by the higher variability of the responses in the 6 m path length trials. Pointing responses for the floor level target trials were not significantly different when comparing IM first versus IM last conditions for either distance (Figure 6b).

Further, when pointing to floor level targets, the correlation between arm azimuth velocity and arm elevation for participants who completed the imagined condition first (*r* = −0.39) was lower than those who completed the imagined condition last (*r* = −0.57). The percentage of trials for which the correlation was significant was 61% for IM first and slightly higher at 66% for IM last. Finally, the RMS values at ±20° arm azimuth for elevated targets were higher for IM first (0.29,±0.11) compared to IM last (0.20,±0.08).

In the post-experimental questionnaire, participants were asked whether, during the imagined condition, they consciously made an effort to increase their arm velocity systematically as a function of their imagined position relative to the target. Only three of the 18 participants indicated that they used such a strategy. When evaluating the pattern of pointing in these three participants it was clear that, even though they all consciously attempted to produce the appropriate pattern of pointing, only one of them was able to approximate the pattern of pointing during actual self-motion.

## Discussion

Overall, this study clearly demonstrates differences between perceived self-motion during actual movement and imagining these same movements during mentally-simulated self-motion. These differences were revealed through the use of continuous pointing, which proved to be a sensitive method for exposing novel features of internally-represented self-motion. In general, it was shown that similar patterns of performance were observed during actual movement, whether blindfolded or sighted. Specifically, pointing responses reflected a clear ability to perceive self-velocity with little systematic error in the absence of vision. Most important, the continuous pointing responses during actual self-motion revealed a characteristic arm trajectory. Specifically, it was shown that arm azimuth velocity increased upon target approach, peaked when aligned with the target and decreased upon target passage. Further, a strong negative correlation was observed between arm azimuth velocity and pointing elevation. These characteristic patterns of arm movements were not as apparent during imagined self-motion. For instance, for elevated targets, arm azimuth velocity tended to quickly reach a plateau and did not change as a function of changing imagined position relative to the spatial image. For floor targets, arm azimuth velocity decelerated more rapidly than that observed for either of the actual self-motion conditions. Further, for floor targets, the correlation between azimuth velocity and elevation was clear for both SW and BW and less so for IM.

There was also some evidence indicating that pointing responses changed as a function of the amount of recent experience with pointing during actual movements. For instance, when pointing to the elevated target in the 5 m trials, the pattern of pointing observed for participants who completed the IM condition last more closely approximated the pattern of pointing observed during actual self-motion (this effect was not as apparent for floor targets). Further, the correlation between arm azimuth velocity and elevation was higher for participants who completed the IM condition last and the RMS errors were lower.

### Differences between Actual Self-Motion and Imagined Self-Motion

Reliable sources of sensory and motor information are not available during imagined movement, which is likely to have caused some of the differences between the pointing movements exhibited during imagined walking and those during actual walking. During actual locomotor movements, sensory and motor information are important for producing an intended movement, receiving feedback about the movement and correcting the movement online if necessary. All such elements are essentially absent during purely imagined movements. That said, locomotor-relevant proprioceptive information is certainly available during imagined movements, except that it indicates that the observer is in a stationary position rather than moving. Consequently, imagined self-motion may suffer from the embodied “grounding” that occurs when an observer knows his/her body to be in a particular point in space relative to the environment (as specified through both proprioceptive and cognitive sources). There is a conflict that is created between one's perceived position in space and one's imagined position in space [56], [57]. Past work has in fact demonstrated that, when asked to take an imagined viewpoint after learning a scene or object layout, observers can more accurately report the direction/location of previously learned targets after a period of disorientation that dissociates the two frames of reference (actual body orientation and imagined orientation) and thus reduces the conflict [58]. There is also evidence that performance on motor-imagery tasks is partially facilitated when the sensory information provided during a concurrently produced behaviour is consistent with the imagined sensory experience, perhaps due to a reduction in this conflict [7], [59].

### Description of Imagined Self-Motion Provided through Continuous Pointing

Despite the obvious differences between imagined and actual movement discussed above, and the distinct and quantifiable differences observed in this study, many research findings obtained using several different experimental approaches (e.g. neuroimaging, autonomic responses, chronometric behavioural tasks) have shown strong (sometimes identical) patterns of responding in both imagined and actual movement tasks. Therefore, it is important to reconcile the different conclusions that are drawn using different tasks and techniques.

In particular, it is important to determine which specific aspects of motor processing are being captured by the various experimental tasks. For instance, most complex movements involve both automatic/unconscious processes as well as higher-level cognitive processes. In other words, performers are explicitly aware of some aspects of their motor behaviour, while other aspects remain implicit. It may be that motor imagery can capture some of the more explicit or cognitive components, while the implicit or automatic processes are more difficult to access offline. The higher-level components might reflect the shared features of actual movement and imagined movement, thus resulting in similar responses on some measures. Differentiating these two aspects of motor behaviour have been considered important for evaluating the true underlying similarities between imagined and actual movements; for example, when assessing what is actually being captured using neuroimaging [50]. The challenge then becomes attempting to access the highly implicit aspects of motor behaviour that occur during a highly explicit activity (i.e., imagery). Therefore, it would be ideal to develop tasks in which specific properties of motor behaviours are not consciously accessible to the performer. This is something that has been more effectively achieved using the continuous pointing method presented here. During this task, there was no awareness from most participants that they needed to move their arm faster as they approached the target and yet they did this effortlessly when walking without visual feedback.

Not only does continuous pointing reveal differences in more implicit aspects of behaviour, but it also provides a more detailed description of precisely which characteristics of actual movement are captured during imagined self-motion. We can therefore consider the different stages of a specific behaviour, including intentional motor planning, online motor output, and ultimately feedback. Whereas the intention to produce a movement is often a conscious decision, once the motor output is produced, it is often controlled automatically (for instance, during reaching). Therefore, while there may be shared elements in the planning of imagined and actual movements, the similarities that occur during the execution of that movement remain relatively unknown. Using a similar strategy of looking specifically at the detailed properties of particular movements, Goodale et al. (1994) showed differences between reaching towards imagined objects and real objects. They reported that the characteristic properties of actual reaching, such as maximum hand velocity, position, and grip aperture, were not the same when the reach was pantomimed [60].

Attempts have also been made to consider how different aspects of motor imagery affect the impact of mental practice on skilled motor performance. Jackson et al. (2001), for instance, differentiate “declarative knowledge”, such as the ability to explicitly describe the sequence of movements and their properties, from “nonconscious processing”, in which the subtle elements of the movements and the coordination of different movements cannot be overtly described [61]. It has been suggested that mental practice mainly facilitates tasks that involve cognitive elements, although it has been shown to be effective for both cognitive and physical tasks [62].

When evaluating the contributions of the continuous-pointing method to the study of imagined self-motion, one of the clearly demonstrated benefits is the amount of information that is obtained during the entire period of imagined motion. In essence, this method provides a window into a level of processing that has been difficult to access in the past. For instance, if we were to revisit the analyses of our imagined walking data and compare them to the most common strategy in the literature of using chronometric measures of imagined motion, the advantages become clear. As a way of evaluating how our own data would be interpreted if we were to reduce it to a discrete duration-based measurement, the mean pointing duration after which the arm reached an angle of −30 degrees (0.7 m past the target) was calculated. For the 6 m path lengths, the imagined walking duration and blindfolded walking duration were 3.4 s and 3.4 s respectively. For the 5 m path length the imagined walking duration and blindfolded walking duration were 2.6 s and 2.7 s respectively. What this tells us, is that if we were to base our conclusions solely on differences in elapsed duration, we would assert that there were almost no differences between imagined and actual motion. This is, of course, very different from our current conclusions based on calculated perceived self-velocity over the entire trajectory. Considering that there are several conflicting findings in the literature regarding whether duration differences are observed between imagined and actual walking conditions [4], [7], [8], our method might help to reconcile these differences, or at least provide more insight into the source of these differences. What is highlighted through this type of comparison is the substantial information loss that comes from many other approaches and how this might then affect the overall interpretation of the data.

### Effect of Recent Experience on Continuous Pointing during Imagined Self-Motion

Also interesting were some of the different trends observed for participants who completed the imagined condition first compared to those who completed it last. Specifically, for the 5 m trials, when pointing to elevated targets participants who completed the imagined condition last showed a pattern of pointing responses that were more similar to pointing during actual movement. Further, the correlation between arm azimuth velocity and elevation were lower when the IM condition was performed first compared to when it was performed last. This synchronous coordination of arm movements reflects a very particular spatial and temporal sequence that is arguably highly implicit and, therefore, particularly revealing.

In contrast to the results seen in the current experiment, previous research using chronometric tasks have shown very little differences when comparing trials in which imagined conditions came first compared to when they came last [7], [49]. Papaxanthis et al. (2002) looked at this issue explicitly and concluded that, at least in terms of temporal processing, performance on the imagined task remained the same as actual movements irrespective of the order in which they were completed [49]. In the research area of mental practice, Courtine et al (2004) also demonstrated that, regardless whether the imagined movement (covert motor practice) and actual movement trials were blocked or interleaved, average imagined movement times did not differ [5]. However, the variability of the imagined movement times decreased if these trials were preceded by the actual movement. Therefore, it was concluded that sensory or motor information can be stored in working memory in a way that facilitates the retrieval of this motor program during covert movement rehearsal. Plumert et al. (2004) examined imagined walking times to targets presented in both a real environment and a virtual simulation of that environment. While no response differences were observed as a function of the different environments (in adults), an environmental order effect was reported. Specifically, participants who imagined walking to virtual targets first and real targets second exhibited a much larger undershoot than those who completed the conditions in the reverse order [40]. Therefore, the original experience of completing the task in one environment carried over to the performance in the following condition. Interestingly, when comparing blind-walking times to imagined walking times, no order effects for these two conditions were observed.

There are several possible explanations as to why differences might be observed as a function of whether the imagined self-motion trials preceded or followed the actual self-motion trials, none of which are mutually exclusive or exhaustive. First, it is possible that over repeated trials of actual self-motion, the capacity to experience a veridical sense of self-motion through motor imagery might have been improved. Second, participants may have gained a conscious awareness of the arm-movement profile that was necessary to accurately represent a specific self-motion profile. Third, pure motor learning based on the learned pattern of arm kinematics may have occurred due to repetitive movements of the arm. This would imply that changes to imagined self-motion did not occur per se, but rather a short-term motor memory system may have come into play. In terms of motor learning, however, it is important to note that the specific pattern of motor behaviour changed frequently during the experiment as a function of the different target relative starting positions, target heights, and target displacements.

While we cannot reconcile these different possibilities here, insight is provided by the fact that participants reported no conscious attempt to reproduce a particular arm velocity profile (thus refuting option two of a cognitive reproduction). Future work will seek to determine whether this experiential effect is a transient change reflecting working memory systems or whether long-term changes in the ability to accurately imagine self-motion occur.

### Conclusions and Broader Implications

Continuous pointing has proven to be a useful tool for carefully defining several unique features of actual self-motion perception and imagined self-motion. In the future, it will be important to further validate this measure by directly comparing these results with those from other measures of self-motion perception and motor imagery. In addition, this method can be used to more systematically evaluate different aspects of spatial cognition, investigate characteristics of multi-sensory integration during locomotion and provide a more objective, quantifiable measure of vection. We have recently introduced this method as a way of more closely investigating inertially-based self-motion perception during complex, passive movements in the absence of vision [17].

The results of the current research are also relevant to investigations studying the impact of mental practice techniques used during the training and evaluation of complex motor behaviours [62], [63]. This method could also have significant implications for methods of motor rehabilitation that rely on therapies utilizing motor imagery [64], [65]. One of the specific concerns of using motor imagery for rehabilitation is that it is difficult to assess whether a patient is actually engaging in imagery in the prescribed way. Continuous pointing could provide an indirect, but explicit, index of both the extent to which the patient is actively engaged in the imagery task and the characteristics of their imagined movements.

## Materials and Methods

### Participants

Twelve participants (5 female and 7 male) between the ages of 22 and 35 (M = 25.67) completed six conditions across two one-hour sessions on separate days. Six additional participants (1 female and 5 male) between the ages of 23 and 30 (M = 25.29) participated only in the BW and IM conditions (see Procedure for full list of conditions completed by each group of participants). All participants had normal or corrected-to-normal vision and were naïve to the purposes of the experiment. All but one participant were right-handed. Participants were recruited from the Max Planck Institute Subject Database and were compensated at a rate of 8 Euros per hour. All participants provided informed written consent before beginning the experiment. This research was performed in accordance with the ethical standards specified by the ethics review board of the Max Planck Institute for Biological Cybernetics and the 1964 Declaration of Helsinki.

### Stimuli/Apparatus

The tracking system recorded the location and orientation of reflective markers mounted on a helmet and on a customized pointing device. The pointing device consisted of an ergonomic grip handle which allowed participants to hold the pointer comfortably while resting their pointing finger in an extended pointing position. Their index finger was fixed to the pointer using medical tape. Each Vicon camera had a resolution of 1280×1024 and the tracking system had an effective sampling rate of 120 Hz. Customized software (veLib, MPI for Biological Cybernetics) recorded the locations of the helmet and pointer approximately 120 times per second.

### Procedure

Six of the participants completed the floor-height target trials first for each condition and the shoulder-height target trials second, another six participants were presented with target heights in the opposite order and the final six participants pointed only to shoulder-height targets. All other starting position parameters were presented in a pseudo-randomized order. For each condition there were 32 trials per participant: 2 travel distances (5 m or 6 m) ×2 target displacements (1.3 m or 3.6 m) ×2 target heights (floor or shoulder; for six participants shoulder height only) ×4 repetitions (1 for each of the four quadrants).

The first 12 participants in this study completed six conditions in total, including sighted walking, blindfolded walking, pointing only upon target passage, sighted passive transport, blindfolded passive transport, and imagined walking. However, only the sighted walking, blindfolded walking, and imagined walking data are presented here. Each participant completed four practice trials of pointing with vision before the experimental trials began.

### Data Analysis

The raw tracking data from the helmet and the pointing device provided continuous rotational and positional information in x, y, z coordinates as participants moved through space. It was assumed that at the beginning of every trial, while under full-cue conditions, participants were pointing as accurately as possible to the target (i.e. they were pointing where they intended to point). This initial pointing error (both azimuth and elevation) was calculated for every trial before any movement was initiated and was then subtracted from all subsequent pointing data in that trial. Mean absolute errors were 19.32 degrees (SD = ±15.79 degrees) for elevation and 3.48 degrees (SD = ±3.60 degrees) for azimuth.

Velocity data were low-pass filtered to reduce noise (first-order Butterworth filter, cutoff frequency of 1 Hz). If, on a given trial, tracking data were not recorded for more than 1/10 of the trial duration (or more than 1/2 of the time needed to establish the initial pointing error), this trial was excluded from the analysis. This occurred, for instance, when the participant's arm was out of range of the minimum number of tracking cameras. Based on these criteria, 5% of the trials were not used. The percentage of unused trials was approximately the same for all conditions. Finally, it is important to note, that while we used this method to measure simple movement trajectories in the current study, applying this method to more complex trajectories introduces additional constraints that must be considered.
